# Is UWLS Really Better for Medical Research?

**DOI:** 10.1002/sim.70411

**Published:** 2026-02-05

**Authors:** Sanghyun Hong, W. Robert Reed

**Affiliations:** ^1^ Department of Economics and Finance and UCMeta University of Canterbury Christchurch New Zealand

**Keywords:** Cochrane Database of Systematic Reviews, fixed effect, medical research, meta‐analysis, pre‐registration, random effects, replication, robustness check, unrestricted weighted least squares

## Abstract

This study evaluates the performance of the Unrestricted Weighted Least Squares (UWLS) estimator in meta‐analyses of medical research. Using a large‐scale simulation approach, it addresses the limitations of model selection criteria in small‐sample contexts. Prior research using the Cochrane Database of Systematic Reviews (CDSR) reported that UWLS outperformed Random Effects (RE) and, in some cases, Fixed Effect (FE) estimators when assessed using AIC and BIC. However, we show that idiosyncratic characteristics of the CDSR datasets, notably their small sample sizes and weak‐signal settings (where key parameters are often small in magnitude), undermine the reliability of AIC and BIC for model selection. Accordingly, we simulate 108 000 datasets mirroring the original CDSR data. This allows us to know the true model parameters and evaluate the estimators more accurately. While all estimators performed similarly with respect to bias and efficiency, RE consistently produced more accurate standard errors than UWLS, making confidence intervals and hypothesis testing more reliable. The comparison with FE was less clear. We therefore recommend continued use of the RE estimator as a reliable general‐purpose approach for medical research, with the choice between UWLS and FE made in light of the likely extent of effect heterogeneity in the data.

## 1 Introduction

Meta‐analysis is a cornerstone of evidence‐based medicine, systematically synthesizing findings from multiple studies to provide robust estimates of treatment effects and resolve inconsistencies across individual investigations. It plays a critical role in informing clinical guidelines and identifying promising avenues for future research. While Fixed Effects and Random Effects models have traditionally been the primary approaches for synthesizing evidence, the statistical landscape of meta‐analysis is continually evolving, with ongoing discussions about the most appropriate models for handling heterogeneity and ensuring reliable inference.

In a recent paper, Stanley et al. [1] studied 67 308 meta‐analyses of medical research from the Cochrane Database of Systematic Reviews (CDSR). They compared three estimators using the model selection criteria AIC and BIC [2, 3, 4, 5]: Fixed Effects (FE), Random Effects (RE), and a variant of the FE estimator they call Unrestricted Weighted Least Squares (UWLS).^1^ Their main conclusion is that UWLS is “a better model of medical research than RE regardless of heterogeneity, number of studies, or the type of outcome.” Their findings with respect to FE were mixed. Accordingly, they recommended that researchers “should routinely report UWLS in all meta‐analyses”.

This study critically investigates the reproducibility and robustness of Stanley et al.'s innovative work. We commend them for providing open access to their data and code, which enables straightforward reproduction of their findings.^2^ However, their conclusions about UWLS hinge critically on the performance of AIC and BIC in small‐sample, weak‐signal settings. The CDSR data used by Stanley et al. have precisely these characteristics: key parameters are often small in magnitude, and the number of estimates per study is limited (median = 5; mean = 8.9). In such conditions, AIC and BIC are well known to select models unreliably [7, 8, 9]. We demonstrate below that such is the case in Stanley et al.'s application.

If AIC and BIC are unreliable, then Stanley et al.'s recommendation for routinely reporting UWLS in meta‐analyses of clinical trials could lead to suboptimal or misleading conclusions. Accordingly, this study reassesses the performance of UWLS and other meta‐analytic estimators under conditions representative of the CDSR, but with the critical advantage of knowing the true underlying model parameters. To achieve this, we simulate 108 000 datasets mirroring the original CDSR data. This allows us to evaluate estimator performance (bias, mean squared error, and coverage probabilities) independent of model selection criteria. This simulation‐based approach provides a robust framework for understanding the true capabilities of each estimator, directly addressing the limitations of AIC/BIC in small samples and weak‐signal environments.

We initiated our study by drafting an analysis plan and circulating it to selected scholars researching in this area, including the authors of Stanley et al. Based on the feedback we received, we modified our analysis plan and registered it on OSF (https://osf.io/wp635).

We have now completed our planned study and analysis. Our main finding is that while UWLS performs similarly to the random‐effects estimator on bias and efficiency, it has substantially poorer coverage and is therefore less reliable for inference. Its performance relative to fixed effects is mixed, with coverage generally worse when true heterogeneity is small and somewhat better when heterogeneity is large. We further show that the very small meta‐analyses characteristic of the Cochrane Database of Systematic Reviews (CDSR) play a central role in the unreliable behavior of AIC and BIC in Stanley et al.'s application. In conclusion, we recommend continued use of the RE estimator as a reliable general‐purpose approach, with the choice between UWLS‐FE and FE made in light of the likely extent of effect heterogeneity in the data.

## 2 Methods

### 2.1 The Estimators to Be Investigated

Let $y_{i}$ denote the estimated effect from primary study i, with reported sampling variance $\sigma_{i}^{2}$. The parameter of interest in our analysis is μ, the population mean of $y_{i}$. All the estimators studied here differ only in how they model the variance of $y_{i}$.

A convenient representation of the respective variance assumptions is provided by the following general specification:

(1) $$y_{i}=\mu +u_{i}+\varepsilon_{i},$$

where $\varepsilon_{i}$ represents sampling error and $u_{i}$ represents between‐study heterogeneity. Conditional on μ, the variance of $y_{i}$ is given by:

(2) $$\mathrm{Var}\left(y_{i}|\mu \right)=\gamma \left(\sigma_{i}^{2}+\tau^{2}\right).$$

In this specification, $\tau^{2}$ is the variance of the individual study true effects, $u_{i}$. The term $\left(\sigma_{i}^{2}+\tau^{2}\right)$ therefore represents the baseline variance of $y_{i}$ arising from the combined contribution of sampling variability and between‐study heterogeneity in true effects. γ is a multiplicative scale factor applied to the baseline variance $\left(\sigma_{i}^{2}+\tau^{2}\right)$. Its interpretation and role differ across estimators and are discussed below. While $y_{i}$ and $\sigma_{i}$ are observed, the parameters μ, $\tau^{2}$, and γ must be estimated.

#### 2.1.1 Fixed Effects (FE)

The fixed‐effects model assumes that all studies report sampling variances that are correctly specified and that there is a single common true effect: $\gamma =1,\tau^{2}=0.$ Accordingly, under FE, all variation in $y_{i}$ is attributed to sampling error:

(3) $$y_{i}=\mu +\varepsilon_{i},\varepsilon_{i}∼N\left(0,\sigma_{i}^{2}\right).$$

The FE model is estimated by inverse‐variance weighted least squares, with weights $w_{i}=1/\sigma_{i}^{2}$. The estimator of μ is the weighted mean ${\hat{\mu }}_{\mathrm{FE}}=\frac{\sum_{i}w_{i}y_{i}}{\sum_{i}w_{i}},$ with variance $1/\sum_{i}w_{i}$.

#### 2.1.2 Random Effects (RE)

The random effects model also assumes that sampling variances are correctly specified but allows for genuine heterogeneity in true effects across studies: $\gamma =1,\tau^{2}>0.$ In this case,

(4) $$y_{i}=\mu +u_{i}+\varepsilon_{i},u_{i}∼N\left(0,\tau^{2}\right),\varepsilon_{i}∼N\left(0,\sigma_{i}^{2}\right).$$

The RE model is estimated by maximum likelihood. The parameters μ and $\tau^{2}$ are jointly estimated and the implied weights take the form $w_{i}=\frac{1}{\sigma_{i}^{2}+\tau^{2}}.$

#### 2.1.3 Unrestricted Weighted Least Squares—Fixed Effects (UWLS‐FE)

Like FE, the UWLS‐FE model maintains the assumption of a common true effect. However, it differs from FE by relaxing the assumption that reported sampling variances fully characterize the dispersion of estimated effects across studies. In particular, UWLS‐FE allows the variance of the sampling error to be multiplicatively scaled, permitting the observed variability of $y_{i}$ to be larger or smaller than implied by $\sigma_{i}^{2}$: γ≠1 and $\tau^{2}=0$. This leads to

(5) $$y_{i}=\mu +\varepsilon_{i},\varepsilon_{i}∼N\left(0,\gamma \sigma_{i}^{2}\right).$$

Unlike $\tau^{2}$, γ does not represent heterogeneity in underlying true effects but rather systematic distortion of reported sampling variances. It has a direct statistical interpretation and is numerically equivalent to the heterogeneity statistic $H^{2}$, defined as the ratio of total variance to sampling variance. Values of γ>1 indicate excess dispersion relative to reported sampling error, while values of γ<1 represent “excessive homogeneity,” meaning that observed variation in effect sizes is smaller than would be expected based on sampling error alone. Stanley et al. argue that such excessive homogeneity may arise from mechanisms such as publication bias, p‐hacking, or selection for statistically significant results.

UWLS‐FE is estimated by weighted least squares with weights $w_{i}=1/\left(\gamma \sigma_{i}^{2}\right)$. Because γ enters multiplicatively, it cancels from the weighted mean, implying that the UWLS‐FE and FE point estimates of μ are identical. However, γ rescales the estimated variance of $\hat{\mu }$ by a factor of γ, thereby affecting standard errors and confidence intervals without altering the point estimate.

#### 2.1.4 Unrestricted Weighted Least Squares—Random Effects (UWLS‐RE)

UWLS‐RE is to RE as UWLS‐FE is to FE: It produces the same point estimate of μ as RE under the two‐step procedure described below, but allows for multiplicative scaling of the variance components. As such it is a natural extension of the UWLS‐FE estimator analyzed by Stanley et al. In the context of the model below, it is characterized by γ≠1 and $\tau^{2}>0$.

(6) $$y_{i}=\mu +u_{i}+\varepsilon_{i},u_{i}∼N\left(0,\gamma \tau^{2}\right),\varepsilon_{i}∼N\left(0,\gamma \sigma_{i}^{2}\right).$$

As before, $\tau^{2}$ captures additive heterogeneity in the underlying true effects across studies, $u_{i}$. The parameter γ acts as a global scale factor governing the overall dispersion of the estimated effects. Allowing γ≠1 relaxes the assumption that the combined contribution of sampling variability and true‐effect heterogeneity $\left(\sigma_{i}^{2}+\tau^{2}\right)$ fully characterizes the variance of $y_{i}$. The RE and UWLS‐FE models arise as special cases when γ=1 or $\tau^{2}=0$, respectively.

UWLS‐RE is estimated using a two‐step procedure.^3^ In the first step, the between‐study heterogeneity variance $\tau^{2}$ is estimated using a conventional random‐effects model. In the second step, $\tau^{2}$ is treated as fixed and incorporated into the variance term, yielding weights $w_{i}=\frac{1}{\gamma \left(\sigma_{i}^{2}+{\hat{\tau }}^{2}\right)}.$ The common effect μ and the multiplicative scale parameter γ are then estimated by weighted least squares using these weights. Because $\tau^{2}$ is fixed in the second step, UWLS‐RE is not estimated by joint maximum likelihood.

While UWLS‐RE is a natural extension of the three estimators considered by Stanley et al. and is useful for robustness checking, its primary role in this analysis is different. Estimation of the UWLS‐RE model allows us to jointly estimate all key parameters of the data‐generating process—μ, γ, and $\tau^{2}$—within a single framework. These parameter estimates provide empirically grounded inputs for the simulation designs developed in the next section.

### 2.2 Using Simulations to Assess Estimator Performance

We use simulation to create meta‐analyses that are representative of the datasets in Stanley et al.'s sample. We then use AIC and BIC to select the “best” models for these simulated datasets. The advantage of using simulated datasets is that we know the true model. Thus we can determine how well AIC and BIC are able to identify the correct model among the four candidates (FE, UWLS‐FE, RE, and UWLS‐RE). After this, we conduct an alternative comparison where we assess the performance of the four estimators with respect to bias, root mean squared error (RMSE), and coverage probabilities.

**FIGURE 1:**
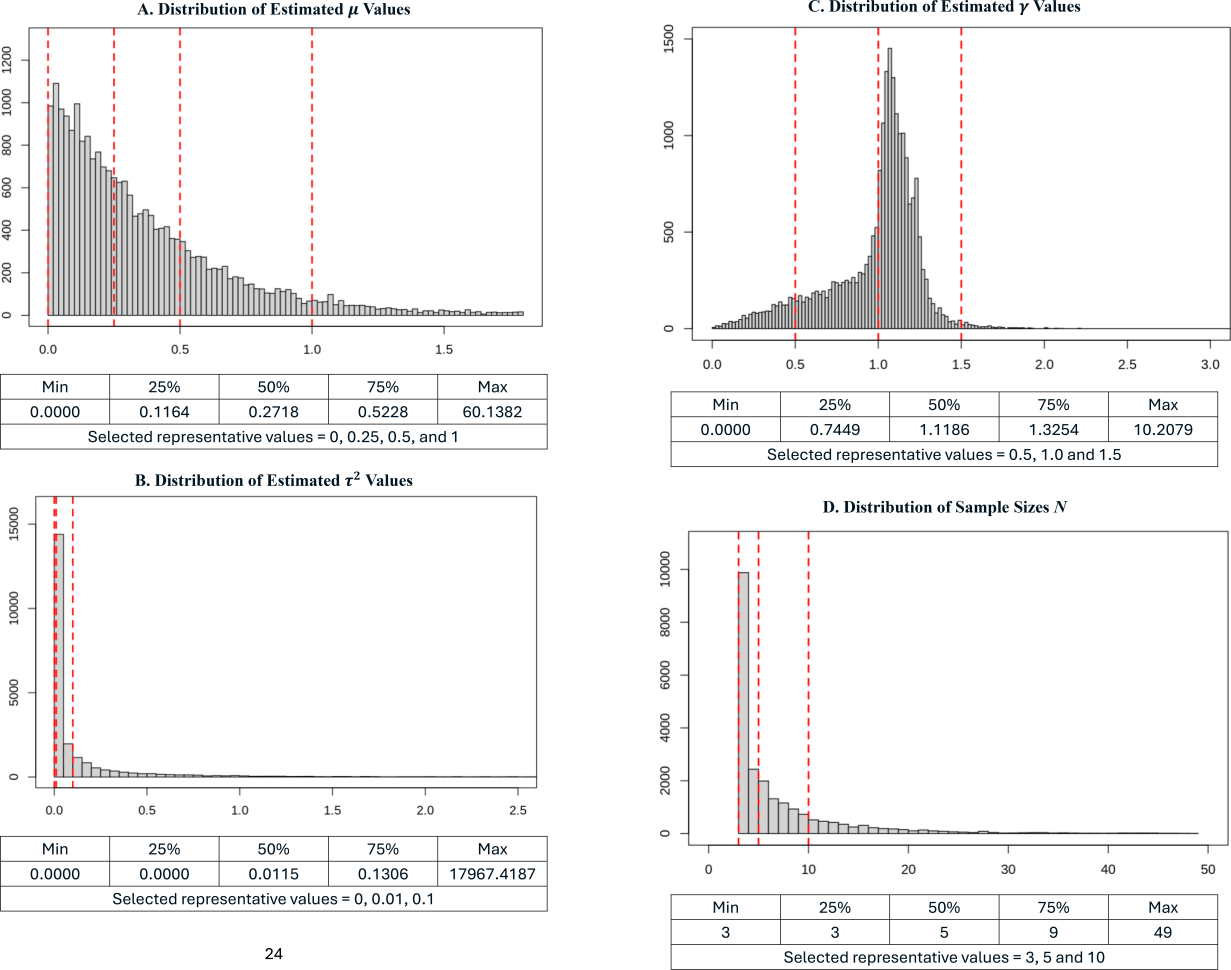
Distribution of estimated parameters. (A) Distribution of estimated μ values. (B) Distribution of estimated $\tau^{2}$ values. (C) Distribution of estimated γ values. (D) Distribution of sample sizes *N*. We used UWLS‐RE to estimate each of the 67 308 meta‐analyses used by Stanley et al. This produced 67 308 estimates of *μ, γ*, and $\tau^{2}$. The figures above report histograms for each of the estimated parameters, along with the number of estimates in each meta‐analysis (*N*). We used these to select representative values for each of the four parameters, given by the red, vertical, dotted lines.

To set the parameters for the DGPs that will produce the meta‐analysis datasets, we exploit the fact that the UWLS‐RE model encompasses the other three estimators. We estimate each of the 67 308 meta‐analysis datasets using UWLS‐RE and record the corresponding estimates of μ, γ, and $\tau^{2}$, along with *N*, the sample size of the respective meta‐analysis. Figure 1 displays the associated histograms and provides selected descriptive statistics.^4^

From these distributions we choose representative values for μ, γ, $\tau^{2}$, and *N*. We selected four values for *μ* (0, 0.25, 0.5, 1), three values for $\tau^{2}$ (0, 0.01, 0.10), three values for *γ* (0.5, 1, 1.5), and three values for *N* (3, 5, 10).^5^ These are reported in Table 1 and are indicated in Figure 1 by the vertical red lines. The respective values give us 108 (= 4 × 3 × 3 × 3) distinct combinations of μ, γ, $\tau^{2}$, and *N*.

**TABLE 1:** Parameter values used to generate simulated meta‐analyses.

Parameter	Values
*μ*	0, 0.25, 0.50, 1.00
*γ*	0.5, 1, 1.5
$\tau^{2}$	0, 0.01, 0.10
*N*	3, 5, 10

*Note: μ* represents the overall population mean effect size (cf. Equations (1), (2), (3), (4), (5), (6)). *γ* represents a global scale factor for effect dispersion (cf. Equations 5 and 6). $\tau^{2}$ represents additive, unobserved effect heterogeneity (cf. Equations 4 and 6). *N* is the number of studies/estimates in the meta‐analysis. The values in the table were selected as being representative as determined by inspection of the histograms in Figure 1.

Each of the 108 combinations of the parameters describes a particular DGP. We can go further and categorize each DGP into one of four types according to the values of γ and $\tau^{2}$.

- *Type 1 (FE)*: (γ = 1, $\tau^{2}$ = 0).
- *Type 2 (RE)*: (γ = 1, $\tau^{2}$ > 0).
- *Type 3 (UWLS‐FE)*: (γ ≠ 1, $\tau^{2}$ = 0).
- *Type 4 (UWLS‐RE)*: (γ ≠ 1, $\tau^{2}$ > 0).

When γ = 1 and $\tau^{2}$ = 0, the UWLS‐RE model collapses to the FE model (cf. Equations 6 and 3). When γ = 1 and $\tau^{2}$ > 0, the UWLS‐RE model collapses to the RE model (cf. Equations 6 and 4) and so on. Table 2 follows through on this by categorizing each of the 108 DGPs by type. Twelve of the DGPs are classified as Type 1 (FE). Twenty‐four are classified as Type 2 (RE). Another 24 are grouped as Type 3 (UWLS‐FE). And 48 belong to Type 4 (UWLS‐RE).

**TABLE 2:** Categorizing all 108 combinations of *μ, ϒ,* $\tau^{2}$, and *N*.

DGP	Values	Number
Type 1 (FE)	μ = (0, 0.25, 0.5, 1), γ = (1), $\tau^{2}$ = (0), *N* = (3, 5, 10)	12
Type 2 (RE)	μ = (0,0.25,0.5,1), γ = (1), $\tau^{2}$ = (0.01, 0.10), *N* = (3, 5, 10)	24
Type 3 (UWLS‐FE)	μ = (0,0.25,0.5,1), γ = (0.5, 1.5), $\tau^{2}$ = (0), *N* = (3, 5, 10)	24
Type 4 (UWLS‐RE)	μ = (0,0.25,0.5,1),γ = (0.5, 1.5), $\tau^{2}$ = (0.01, 0.10), *N* = (3, 5, 10)	48

*Note:* There are 108 unique combinations of *μ, γ*, $\tau^{2}$, and *N*. Each combination defines a specific DGP according to Equations (1) and (2) through (6). This table categorizes the 108 DGPS according to the type of heterogeneity they have, as represented by γ and $\tau^{2}$. Type 1 (FE) heterogeneity is represented by (γ = 1, $\tau^{2}$ = 0). Type 2 (RE) heterogeneity is represented by (γ = 1, $\tau^{2}$ > 0). Type 3 (UWLS‐FE) heterogeneity is represented by (γ ≠ 1, $\tau^{2}$ = 0). Type 4 (UWLS‐RE) is represented by (γ ≠ 1, $\tau^{2}$ > 0).

Categorizing the different DGPs allows us to check whether BIC and AIC are able to correctly identify the underlying model type/DGP. This is the basis of Stanley et al.'s conclusion that since UWLS‐FE produced lowest BIC/AIC values, it “best represented” medical research.

### 2.3 Simulating the Data

To complete the DGPs in Equations (1) and (2) through (6), we estimated representative $\sigma_{i}$ values for each of the three sample sizes. These are reported in Table 3. Given values for *μ, γ*, $\tau^{2}$, *N*, and the $\left(\sigma_{1},\sigma_{2},\ldots ,\sigma_{N}\right)$ values from Table 3, we use Equation (6) to simulate 1000 individual meta‐analysis datasets for each of the 108 DGPs, producing a total of 108 000 datasets. We then estimate *μ*, its standard error, AIC, and BIC for each dataset using each of the four estimators (FE, RE, UWLS‐FE, and UWLS‐RE). From these 108 000 sets of estimates, we calculate average AIC and BIC values, bias, RMSE, and coverage probabilities for each estimator.

**TABLE 3:** $\sigma_{i}$ values used for simulated meta‐analyses of sample sizes *N* = 3, 5, and 10.

Average $\sigma_{i}$ values (lowest to highest)	$\sigma_{i}$ values used in simulations
*N* = 3	
$\sigma_{1}$ = 0.2845	$\sigma_{1}$ = 0.30
$\sigma_{2}$ = 0.3939	$\sigma_{2}$ = 0.40
$\sigma_{3}$ = 0.5343	$\sigma_{3}$ = 0.50
*N* = 5	
$\sigma_{1}$ = 0.2347	$\sigma_{1}$ = 0.20
$\sigma_{2}$ = 0.3143	$\sigma_{2}$ = 0.30
$\sigma_{3}$ = 0.3913	$\sigma_{3}$ = 0.40
$\sigma_{4}$ = 0.4841	$\sigma_{4}$ = 0.50
$\sigma_{5}$ = 0.6167	$\sigma_{5}$ = 0.60
*N* = 10	
$\sigma_{1}$ = 0.1877	$\sigma_{1}$ = 0.15
$\sigma_{2}$ = 0.2378	$\sigma_{2}$ = 0.20
$\sigma_{3}$ = 0.2815	$\sigma_{3}$ = 0.25
$\sigma_{4}$ = 0.3221	$\sigma_{4}$ = 0.30
$\sigma_{5}$ = 0.3649	$\sigma_{5}$ = 0.35
$\sigma_{6}$ = 0.4081	$\sigma_{6}$ = 0.40
$\sigma_{7}$ = 0.4588	$\sigma_{7}$ = 0.45
$\sigma_{8}$ = 0.5152	$\sigma_{8}$ = 0.50
$\sigma_{9}$ = 0.5862	$\sigma_{9}$ = 0.55
$\sigma_{10}$ = 0.6961	$\sigma_{10}$ = 0.60

*Note:* As is clear from Equations (1) and (2) to (5), in order to simulate meta‐analyses datasets, in addition to *μ, γ*, $\tau^{2}$, and *N*; we need to specify $\sigma_{i}$ for each of the observations in a given, simulated, meta‐analysis dataset. As discussed in the text, we do that by averaging the $\sigma_{i}$ values reported in the 67 308 meta‐analysis datasets in Stanley et al.

## 3 Results Based on the Pre‐Registration Plan

### 3.1 Comparison With Stanley et al.'s AIC and BIC Results

Table 4 demonstrates that our simulated datasets, designed to represent Stanley et al.'s datasets, produce the same conclusions with respect to AIC and BIC. The top panel (Panel A) reports the average AIC and BIC values for all 108 000 meta‐analyses. They are ranked from “best” (smallest value) to “worst” (highest value). Both AIC and BIC select UWLS‐FE (cf. gray‐highlighted cells) as a “better model of medical research” than its competitors.

**TABLE 4:** AIC and BIC model selection: Full sample of 108 000 simulated datasets.

AIC	BIC
*A. Average IC values*	
UWLS‐FE (7.892)	UWLS‐FE (7.232)
FE (8.181)	FE (7.851)
RE (9.337)	RE (8.677)
UWLS‐RE (9.863)	UWLS‐RE (8.873)
*B. Rate that UWLS‐FE is preferred to RE*	
83.7%	83.7%
From Stanley et al. [1]: “The probability that a randomly selected systematic review from the CDSR would favor UWLS over RE is 79.4%” (Abstract)	
*C. Rate that UWLS‐FE is preferred to FE*	
36.2%	41.6%
From Stanley et al. [1]: “When comparing FE and UWLS, BIC favors FE slightly more often (51.0%)” (Page 56)	

*Note:* The top panel (Panel A) reports the average AIC and BIC values for all 108 000 meta‐analyses. They are ranked from “best” (smallest value) to worst (highest value). Both AIC and BIC select UWLS‐FE (cf. gray‐highlighted cells) as a “better model of medical research” than its competitors. Panel B reports that, across the 108 000 meta‐analyses, UWLS‐FE is favored over RE 83.7% of the time. This is close to the 79.4% rate reported by Stanley et al. The last panel reports the rate that UWLS‐FE is favored over FE. Stanley et al. do not report favoring rates for AIC, but for BIC they find that UWLS‐FE is preferred to FE 49.0% of the time. We obtain a favoring rate of 41.6%. These results demonstrate that our simulated datasets, designed to be representative of Stanley et al.'s, produce the same general conclusions as they report.

Panel B reports that, across the 108 000 meta‐analyses, UWLS‐FE is favored over RE 83.7% of the time. This is close to the 79.4% rate reported by Stanley et al. We note that we would not expect the numbers to be identical since the latter comes from the 67 308 meta‐analyses in Stanley et al.'s dataset, while our number is based on the 108 000 simulated datasets designed to be representative of Stanley et al.'s data. Nevertheless, they are quite close.

The last panel reports the rate that UWLS‐FE is favored over FE. Stanley et al. do not report favoring rates for AIC, but for BIC they find that UWLS‐FE is preferred to FE 49.0% of the time. We obtain a favoring rate of 41.6%. Again, not identical, but close. In conclusion, our simulated datasets, designed to be representative of Stanley et al.'s, produce the same general conclusions as they report.

### 3.2 AIC and BIC Do a Poor Job of Selecting the Correct DGP

The first indication that there is a problem is provided by Table 5. This repeats the analysis of Panel A in Table 4, but it separates the 108 000 meta‐analyses across the four types of DGPs. The advantage of using simulated data is that we know the correct model. Table 5 shows us how successful AIC and BIC are in selecting the correct model. Consistent with Table 4, we see that the UWLS‐FE model (gray‐highlighted) is frequently selected as the best model across the four model types/DGPs, even when UWLS‐FE is not the true model.

**TABLE 5:** AIC and BIC model selection by type of DGP.

AIC	BIC
*A. TRUE MODEL: Type 1 (FE)—12 DGPs*	
FE (6.708)	FE (6.378)
UWLS‐FE (7.153)	UWLS‐FE (6.494)
RE (8.535)	RE (7.876)
UWLS‐RE (9.144)	UWLS‐RE (8.155)
***AIC = 73.9%***	***BIC = 68.2%***
*B. TRUE MODEL: Type 2 (RE)—24 DGPs*	
FE (8.900)	UWLS‐FE (8.460)
UWLS‐FE (9.120)	FE (8.570)
RE (10.020)	RE (9.361)
UWLS‐RE (11.037)	UWLS‐RE (10.047)
***AIC = 8.7%***	***BIC = 9.4%***
*C. TRUE MODEL: Type 3 (UWLS‐FE)—24 DGPs*	
UWLS‐FE (6.317)	UWLS‐FE (5.657)
FE (6.747)	FE (6.417)
RE (8.346)	UWLS‐RE (7.356)
UWLS‐RE (8.378)	RE (7.719)
***AIC = 39.6%***	***BIC = 44.8%***
*D. TRUE MODEL: Type 4 (UWLS‐RE)—48 DGPs*	
UWLS‐FE (8.251)	UWLS‐FE (7.591)
FE (8.907)	FE (8.578)
RE (9.677)	RE (9.017)
UWLS‐RE (10.215)	UWLS‐RE (9.226)
***AIC = 0.0%***	***BIC = 0.0%***

*Note:* Table 5 repeats the analysis of Table 4, but it separates the 108 000 meta‐analyses across the four types of DGPs. The purpose of the table is to investigate how often AIC and BIC select the correct model. The top panel (Panel A) generates 12 000 meta‐analysis datasets (1000 for each of the 12 FE DGPs) and calculates AIC and BIC values for each of the four models for every dataset. The table reports the average AIC and BIC values for each model. For the FE DGPs of Panel A, average AIC and BIC values are lowest for the FE model, indicating that the FE model is the correct one. Panel B repeats the analysis for all the RE DGPs. However, average AIC and BIC values incorrectly select the FE and UWLS‐FE models, respectively. The correct model (= RE) is ranked third. In Panel C, average AIC and BIC values select the correct model (= UWLS‐FE). But in Panel D, the correct model (= UWLS‐RE) is ranked last. The gray‐shaded cells highlight that AIC and BIC often select the UWLS‐FE model as the best or close to the best, even when it is not the correct model.

AIC and BIC get off to a promising start in Panel A. This panel reports the results for all meta‐analyses where the FE model is the true model. If AIC and BIC are performing well, then we should find, on average, that FE models receive lower values than the other models. In fact, that is what we see. FE has the lowest average AIC and BIC scores, with UWLS‐FE ranked second best. The last row underscores this performance. It reports the success rates of AIC and BIC in selecting the correct (= FE) model. The two information criteria (IC) select the correct model (out of four) 73.9% and 68.2% of the time.

Panel B repeats the analysis for all those meta‐analyses whose DGPs correspond to the RE model. In contrast to the previous case, the two IC do not perform well. According to AIC and BIC, both FE and UWLS‐FE represent the research better than the RE model, even though the data are, in fact, generated by a RE model. The last row underscores their poor performance. AIC and BIC select the correct model only 8.7% and 9.4% of the time.

Similar results obtain for the last two types of DGPs. While the average AIC and BIC values correctly select the UWLS‐FE model when it is the true one (Panel C), they incorrectly select UWLS‐FE when the true model is UWLS‐RE (Panel D). In the latter case, both IC rank UWLS‐RE last, despite it being the true model. While AIC and BIC achieve better‐than‐chance success rates in Panel C (39.6% and 44.8% compared to 25% from random chance), their performance is dismal in Panel D. The two ICs almost never select the correct model (0.0% in either case).

### 3.3 AIC and BIC Perform Poorly Because the Sample Sizes Are Small

As noted above, the justification for using AIC and BIC to select the “best” model is based on their asymptotic properties. But the sample sizes in the CDSR meta‐analyses are very small. The median sample size of a CDSR meta‐analysis is 5 estimates. The average sample size is 8.9 estimates. Table 6 shows how the success rate of AIC and BIC in selecting the correct model is affected by sample size.

**TABLE 6:** Accuracy of AIC and BIC in identifying correct DGP by sample size.

	FE (4 DGPs)		RE (8 DGPs)		UWLS‐FE (8 DGPs)		UWLS‐RE (16 DGPs)	
Sample size	AIC (%) (1)	BIC (%) (2)	AIC (%) (3)	BIC (%) (4)	AIC (%) (5)	BIC (%) (6)	AIC (%) (7)	BIC (%) (8)
3	67.5	53.2	3.2	5.2	39.1	52.6	0.0	0.0
5	75.6	69.8	7.8	8.9	36.9	42.6	0.0	0.0
10	78.6	81.6	15.2	13.9	43.0	39.2	0.0	0.0
100	81.2	96.4	51.0	45.7	86.6	80.8	4.2	0.1
500	81.7	98.6	70.6	57.5	98.5	98.5	22.7	11.3
1000	81.1	98.8	81.5	65.1	99.8	99.8	25.2	23.2
5000	80.4	99.6	96.6	96.2	100	100	48.9	25.0

*Note:* Table 6 generates 1000 meta‐analysis datasets for each of the 36 DGPs having a given sample size. The 36 DGPs are divided into the four types of DGPs (FE, RE, UWLS‐FE, and UWLS‐RE). The numbers in the table represent how frequently AIC and BIC select the correct model (out of the four possibilities). For example, in the first row (Sample Size = 3), of the 4000 FE meta‐analysis datasets consisting of three estimated effects each, AIC correctly selected the FE model over the other three models 67.5% of the time. BIC correctly selected the FE model 53.2% of the time. For the 8000 datasets consisting of 3 estimated effects each and generated from a RE DGP, AIC correctly selected the RE model only 3.2% of the time, and BIC only 5.2% of the time. Subsequent rows report how often AIC and BIC selected the correct model as the number of estimated effects in each meta‐analysis increases from 3 to 5 to 10 to 100 … to 5000. We note that the median sample size in Stanley et al.'s data is 5, and the average sample size is 8.9 estimated effects.

To generate the results in Table 6, we took the 36 DGPs as defined by the respective combinations of μ, γ, and $\tau^{2}$, and we generated 1000 meta‐analysis datasets for each DGP for a given sample size.^6^ We started with 3 estimated effects in each meta‐analysis dataset, then increased sample size to 5, to 10, to 100, all the way up to 5000.^7^ For each sample size, the 36 000 meta‐analysis datasets were separated by the type of DGP that generated them (4000 FE datasets, 8000 RE datasets, 8000 UWLS‐FE datasets, and 16 000 UWLS‐RE datasets).

For example, when all meta‐analyses consisted of three estimated effects, and the datasets were generated from one of the four FE DGPs, AIC correctly selected the FE model 67.5% of the time. BIC correctly selected the FE model 53.2% of the time. When we repeated the experiment, increasing the size of the meta‐analysis datasets to 5, AIC and BIC improved their success rates to 75.6% and 69.8%, respectively. As the sample size increased further, both AIC and BIC improved in their ability to select the correct DGP.

AIC's performance stabilized at around 80% when sample sizes reached 100 estimated effects per meta‐analysis, whereas BIC's accuracy improved to nearly 100% with 5000 estimates per dataset. This is consistent with the fact that AIC does not necessarily select the true model as *n* → ∞. Instead, it favors models that optimize predictive accuracy. In contrast, BIC has the asymptotic property of selecting the correct model with 100% success as the sample size becomes infinitely large.

The results from Table 6 are consistent with what we found in Table 5. AIC and BIC do a relatively good job of selecting the correct model when the data are generated by a FE or UWLS‐FE DGP. But they do a poor job of selecting the correct model when the DGP is a RE or UWLS‐RE DGP and sample sizes are small (e.g., 3, 5, or 10). AIC and BIC tend to select the UWLS‐FE and FE models even when the correct model is RE or UWLS‐RE. Table 6 demonstrates that the CDSR meta‐analyses require sample sizes of at least 1000 to consistently and accurately select random‐effects models. The propensity of AIC and BIC to incorrectly select UWLS‐FE and FE over RE models for the smaller‐sized meta‐analyses studied by Stanley et al. provides an explanation for why they obtained their result.

### 3.4 Measures of Estimator Performance

Having demonstrated that AIC and BIC are not reliable for selecting the best model because of the small sample sizes of the CDSR dataset, we now compare the different estimators using measures of bias, RMSE, and coverage rates. Table 7A reports the results.

**TABLE 7A:** Estimator performance.

Bias	RMSE	Coverage rates (%)
*A. All—108 DGPs*		
FE/UWLS‐FE (−0.0004)	FE/UWLS‐FE (0.1792)	RE (93.4)
		FE (89.9)
RE/UWLS‐RE (−0.0004)	RE/UWLS‐RE (0.1795)	UWLS‐RE (86.0)
		UWLS‐FE (85.6)
*B. TRUE MODEL: Type 1 (FE)—12 DGPs*		
FE/UWLS‐FE (−0.0009)	FE/UWLS‐FE (0.1554)	FE (95.1)
		RE (95.8)
RE/UWLS‐RE (−0.0011)	RE/UWLS‐RE (0.1582)	UWLS‐FE (87.3)
		UWLS‐RE (87.2)
*C. TRUE MODEL: Type 2 (RE)—24 DGPs*		
FE/UWLS‐FE (−0.0018)	RE/UWLS‐RE (0.1953)	RE (91.6)
		FE (87.1)
RE/UWLS‐RE (−0.0020)	FE/UWLS‐FE (0.1980)	UWLS‐RE (85.3)
		UWLS‐FE (84.8)
*D. TRUE MODEL: Type 3 (UWLS‐FE)—24 DGPs*		
RE/UWLS‐RE (0.0006)	FE/UWLS‐FE (0.1489)	FE (94.5)
		RE (96.0)
FE/UWLS‐FE (0.0008)	RE/UWLS‐RE (0.1535)	UWLS‐FE (87.0)
		UWLS‐RE (87.0)
*E. TRUE MODEL: Type 4 (UWLS‐RE)—48 DGPs*		
FE/UWLS‐FE (0.0007)	RE/UWLS‐RE (0.1900)	RE (92.6)
		FE (87.6)
RE/UWLS‐RE (0.0008)	FE/UWLS‐FE (0.1908)	UWLS‐RE (85.4)
		UWLS‐FE (84.8)

*Note:* The table represents the results of estimating 108 000 meta‐analyses. Each of the 108 DGPs was used to simulate 1000 meta‐analysis datasets. For each of these datasets, the mean treatment effect was estimated using each of four estimators: FE, RE, UWLS‐FE, and UWLS‐RE, and the corresponding BIC and AIC values were calculated and recorded. These were then aggregated by DGP, and bias, RMSE, and coverage rates were calculated. These average values were themselves then averaged for the full sample of 108 DGPs, and for each of the four subgroups of types of heterogeneity models. Shaded cells highlight the UWLS‐FE estimator.

There are five panels. The top panel reports the pooled results from the 108 000 simulated meta‐analyses. The next four panels allocate the meta‐analyses across DGP types (FE, RE, UWLS‐FE, and UWLS‐RE). The first two columns of the table report bias and RMSE. As noted previously, FE and UWLS‐FE produce identical coefficients, differing only in their standard errors. As a result, they will produce identical bias and RMSE values. Accordingly, we combine them for the purposes of reporting bias and RMSE results. For the same reason, we combine RE and UWLS‐RE when reporting on bias and RMSE.

The average bias for the FE/UWLS‐FE estimator across all 108 000 meta‐analyses is −0.0004. The average bias for the RE/UWLS‐RE estimator is identical to the fourth decimal place. The average RMSE values for FE/UWLS‐FE and RE/UWLS‐RE are also extremely close, identical through the first three decimal places. As a point of reference, it is useful to recall that the DGPs assume four values for μ: 0.0, 0.25, 0.5, and 1.0. Thus, the differences in bias and RMSE are not practically meaningful and likely due to simulation sampling error.

In contrast, we see substantial differences in the coverage rates of the four estimators. For the RE estimator, 93.4% of the 95% confidence intervals for μ include the true value of μ. For the FE estimator, 89.9% of the 95% confidence intervals include the true value. The UWLS‐FE estimator, highlighted in gray in the table, performed worst. Only 85.6% of its 95% confidence intervals include the true value of μ.

This pattern is generally repeated when we separate the meta‐analyses by DGP type. Bias and RMSE are always very close; too close to recommend any one estimator over the others. However, there are substantial differences in coverage rates. For some DGP types, RE produces confidence intervals closest to their nominal values of 95% (Panel C, Panel E). In other cases, FE is best (Panel B, Panel D). In no case does UWLS‐FE perform best, always ranking third or last.^8^ We also note that UWLS‐RE, while sometimes performing better than UWLS‐FE, is always outperformed by FE and RE.

## 4 Going Beyond the Pre‐Registration Plan

Up to this point we have followed the analysis plan that we registered at OSF. However, after seeing these results, it occurred to us that we have not made the best possible case in support of Stanley et al.'s findings. Specifically, the results in Table 7A give equal weight to each of the 108 DGPs. But suppose the meta‐analyses in Stanley et al. were not proportionately represented by these DGPs? Suppose some DGPs were more representative than others? And suppose for these DGPs, the UWLS‐FE had best estimator performance?

To address this concern, we used Mahalanobis distance matching [12, 13] to match each meta‐analysis to a unique DGP based on their μ, γ, $\tau^{2}$, and *N* values. The results from the 108 DGPs/experiments were then reweighted using the respective number of matches.^9^ Table 7B presents the results.

**TABLE 7B:** Estimator performance (weighted by DGP type).

Bias	RMSE	Coverage rates (%)
*A. All—108 DGPs*		
RE/UWLS‐RE (−0.0007)	FE/UWLS‐FE (0.1655)	RE (94.4)
		FE (91.1)
FE/UWLS‐FE (−0.0008)	RE/UWLS‐RE (0.1656)	UWLS‐RE (86.5)
		UWLS‐FE (86.1)
*B. TRUE MODEL: Type 1 (FE)—12 DGPs*		
FE/UWLS‐FE (−0.0005)	FE/UWLS‐FE (0.1499)	FE (95.3)
		RE (95.9)
RE/UWLS‐RE (−0.0009)	RE/UWLS‐RE (0.1525)	UWLS‐FE (87.8)
		UWLS‐RE (87.7)
*C. TRUE MODEL: Type 2 (RE)—24 DGPs*		
FE/UWLS‐FE (−0.0017)	RE/UWLS‐RE (0.1635)	RE (91.2)
		UWLS‐RE (88.2)
RE/UWLS‐RE (−0.0019)	FE/UWLS‐FE (0.1681)	UWLS‐FE (87.2)
		FE (84.9)
*D. TRUE MODEL: Type 3 (UWLS‐FE)—24 DGPs*		
RE/UWLS‐RE (−0.0004)	FE/UWLS‐FE (0.1329)	FE (97.4)
		RE (97.9)
FE/UWLS‐FE (−0.0004)	RE/UWLS‐RE (0.1350)	UWLS‐FE (85.9)
		UWLS‐RE (85.9)
*E. TRUE MODEL: Type 4 (UWLS‐RE)—48 DGPs*		
RE/UWLS‐RE (0.0002)	RE/UWLS‐RE (0.2590)	RE (87.8)
		UWLS‐RE (84.6)
FE/UWLS‐FE (−0.0006)	FE/UWLS‐FE (0.2604)	UWLS‐FE (83.6)
		FE (79.1)

*Note:* This table is identical to Table 7A except that the results have been weighted by the number of DGPs per type using the Mahalanobis matching procedure described in Section 4. As before, the UWLS‐FE estimator is highlighted in grey.

The results are little changed from Table 7A. Bias and RMSE values are again all very close, so that there is little to distinguish the four estimators on these performance dimensions. This is true both for the full sample (Panel A) and for each of the four subsamples (Panels B through E). The only distinguishing performance measure is the coverage probabilities.

As before, in the full sample of 108 000 meta‐analyses (Panel A), UWLS‐FE has the worst coverage performance. Only 86.1% of its 95% confidence intervals include the true value of μ. This stands in contrast to RE, which again has the best overall performance in the full sample. It has a coverage rate of 94.4%, very close to its nominal value. Across the four subsamples, RE always outperforms UWLS‐FE on coverage rates. While FE dominates UWLS‐FE in the panels with no effect heterogeneity (Types 1 and 3), UWLS‐FE produces better coverage rates in the panels with effect heterogeneity (Types 2 and 4).

## 5 Discussion

Stanley et al. [1] conclude, based on AIC and BIC comparisons in 67 308 meta‐analyses from the Cochrane Database of Systematic Reviews (CDSR), that the UWLS‐FE estimator “better represents medical research” than the Random Effects (RE) estimator, and in some cases even Fixed Effects (FE). We are able to reproduce these results exactly using their data and code. However, their conclusion rests critically on the assumption that AIC and BIC are reliable indicators of model performance in this setting. Our results suggest otherwise. Because meta‐analyses in medicine routinely inform clinical guidelines, regulatory decisions, and patient care, the question of which estimator to use is not merely technical—it has direct implications for the conclusions drawn from evidence syntheses.

The meta‐analyses in the CDSR are not only small (median *N* = 5), but also frequently weak‐signal settings, where both the extent of effect heterogeneity (τ^2^) and the overall effect size (μ) are often close to zero. In such environments, sampling noise is large relative to the underlying signal. Under these conditions, AIC and BIC are known to perform poorly and tend to prefer simpler models, even when more complex models better represent the data‐generating process. This is consistent with our finding that UWLS‐FE is often selected as “best” by AIC/BIC in the CDSR, even in cases where the true model includes nontrivial between‐study variation.

By simulating 108 000 datasets calibrated to the CDSR, we were able to evaluate estimator performance under known truth. Across these simulations, FE, RE, UWLS‐FE (and UWLS‐RE) produced very similar point estimates, showing little difference in bias or efficiency. The meaningful differences arose in standard error accuracy and coverage. The RE estimator consistently produced coverage rates closest to nominal levels across all data‐generating processes. When true heterogeneity was negligible (*τ* ^2^ ≈ 0), FE provided more accurate coverage than UWLS‐FE. However, when heterogeneity was present (*τ* ^2^ > 0), UWLS‐FE achieved better coverage than FE. Even in these cases, though, UWLS‐FE did not match the performance of the RE estimator, which remained the most reliable for inference.

In short, the apparent strong performance of UWLS‐FE in Stanley et al. reflects the limitations of AIC and BIC in small‐sample, weak‐signal meta‐analysis rather than any substantive advantage of the estimator itself. Because meta‐analysis plays a central role in evidence‐based medicine, careful estimator selection is essential: inappropriate model choice can overstate or understate uncertainty and thereby influence subsequent scientific and clinical decisions. We therefore recommend continued use of the random‐effects estimator as a reliable general‐purpose approach, with the choice between UWLS‐FE and fixed effects made in light of the likely extent of effect heterogeneity in the data.

## Funding

This research was supported by the Czech Science Foundation (21‐09231S).

## Disclosure

Pre‐registration posted here: https://osf.io/wp635.

## Conflicts of Interest

The authors declare no conflicts of interest.

## Data Availability

Data and code to reproduce the results in this paper are posted here: https://osf.io/3wfqe/.

## ENDNOTES

^1^
The UWLS estimator was first suggested by Thompson and Sharp [6].

^2^
See Appendix A.

^3^
We originally attempted to estimate $\tau^{2}$ jointly with μ and γ using maximum likelihood, but convergence was an issue. Further, estimates were unstable and unreliable. As a result, we moved to a two‐stage estimation procedure.

^4^
All estimation was conducted in R [10]. To estimate the UWLS‐RE model, we used a combination of the lm() function and the rma() function from the metafor package [11]. Specifically, we first estimate a random‐effects model using rma() and obtain the corresponding estimate of τ^2^. We then use this estimate to construct the inverse‐variance weights for the subsequent weighted least squares regression implemented via lm(). The parameter γ is obtained from the lm() output as the square of the reported residual standard error.

^5^
In selecting representative values for μ, we took absolute values of all estimates.

^6^
There are 108 DGPs in total; 36 with sample size equal to 3, 5, and 10, respectively (cf. Table 2). Thus, for a given sample size, there are 36 DGPs.

^7^
For *N* > 10, each $\sigma_{i}$ was replicated *k* times, where $k=\frac{N}{10}$. As an example, if *N* were equal to 20, ${\left\{\sigma_{i}\right\}}_{i=1\;\text{to}\;20}=\left\{\sigma_{1},\sigma_{1},\sigma_{2},\sigma_{2},\ldots ,\sigma_{10},\sigma_{10},\;\right\}$. In this way, standard errors were generated for *N = 100, 500, 1000*, and *5000*.

^8^
Table 7A uses *z*‐critical values when calculating confidence intervals for the FE and RE estimators (*t‐*critical values are used for the two UWLS estimators). As a robustness check, we investigated whether relative rankings of coverage rates would be affected if one also used *t*‐critical values for the FE and RE confidence intervals. The results are reported in Appendix B. The relative rankings of UWLS‐FE are unchanged.

^9^
The top 40 DGPs by Mahalanobis weights are reported in Appendix C.

## Appendix A Reproduction of Table 1 in Stanley et al. [1]

**Table:**

	Stanley et al. [1]		Our replication results	
Subgroups	Number of meta‐analyses	Prop. favoring UWLS	Number of meta‐analyses	Prop. favoring UWLS
*Heterogeneity by Tau*				
*τ* = 0	29 845	100%	29 845	100.0%
0 < *τ* ≤ 0.2	19 958	71.8%	19 958	71.8%
*τ* > 0.2	17 505	52.9%	17 505	52.9%
*Heterogeneity by γ*				
No heterogeneity: γ ≤ 1	30 827	99.2%	30 827	99.2%
Small: 1 < γ < 2	17 417	66.7%	17 417	66.7%
Moderate: 2 ≤ γ < 4	10 919	58.8%	10 919	58.8%
Large: γ ≥ 4	8145	59.3%	8145	59.3%
*Number of studies (tertiles)*				
Small: (3 ≤ κ ≤ 4)	26 834	59.6%	26 834	86.6%
Medium: (5 ≤ κ ≤ 8)	20 609	78.4%	20 609	79.9%
Large: (9 ≥ κ)	19 865	69.1%	19 865	69.1%
*Outcome measure*				
Continuous	22 815	76.8%	22 815	76.8%
Dichotomous	44 493	80.7%	44 493	80.7%

*Note:* This table demonstrates that we are able to exactly reproduce Stanley et al.'s results using the data and code they provide.

## Appendix B Table 7A Robustness Check: Coverage Rates

**Table:**

Original	FE/RE use t‐stat^a^
*A. All—108 DGPs*	
RE (94.4)	RE (98.3)
FE (91.1)	FE (96.4)
UWLS‐RE (86.5)	UWLS‐RE (86.5)
UWLS‐FE (86.1)	UWLS‐FE (86.1)
*B. TRUE MODEL: Type 1 (FE)—12 DGPs*	
FE (95.3)	FE (99.2)
RE (95.9)	RE (99.0)
UWLS‐FE (87.8)	UWLS‐FE (87.8)
UWLS‐RE (87.7)	UWLS‐RE (87.7)
*C. TRUE MODEL: Type 2 (RE)—24 DGPs*	
RE (91.2)	RE (95.5)
UWLS‐RE (88.2)	FE (95.5)
UWLS‐FE (87.2)	UWLS‐RE (88.2)
FE (84.9)	UWLS‐FE (87.2)
*D. TRUE MODEL: Type 3 (UWLS‐FE)—24 DGPs*	
FE (97.4)	FE (98.5)
RE (97.9)	RE (99.0)
UWLS‐FE (85.9)	UWLS‐FE (85.9)
UWLS‐RE (85.9)	UWLS‐RE (85.9)
*E. TRUE MODEL: Type 4 (UWLS‐RE)—48 DGPs*	
RE (87.8)	FE (95.2)
UWLS‐RE (84.6)	RE (98.0)
UWLS‐FE (83.6)	UWLS‐RE (84.6)
FE (79.1)	UWLS‐FE (83.6)

^a^ The original analysis of Table 7A used *z*‐critical values when calculating confidence intervals for the FE and RE estimators (the two UWLS estimators were already using *t*‐critical values). As a robustness check, we investigate whether relative rankings of coverage rates are affected if one instead uses *t*‐critical values. As before, the UWLS‐FE estimator is highlighted in grey.

## Appendix C Top 40 Models: Mahalanobis Weights

**Table:**

Model type	μ	$\tau^{2}$	γ	N	Percent	Cum. percent
UWLS‐FE	0	0	0.5	3	9.8%	9.8%
UWLS‐FE	0	0	0.5	5	8.6%	18.4%
UWLS‐FE	0	0	0.5	10	6.0%	24.4%
FE	0	0	1	10	4.5%	28.8%
FE	0	0	1	5	4.3%	33.1%
RE	0	0.1	1	10	4.1%	37.2%
RE	0	0.01	1	10	3.8%	40.9%
FE	0	0	1	3	3.2%	44.2%
UWLS‐FE	0.25	0	0.5	3	2.8%	47.0%
UWLS‐FE	0	0	1.5	3	2.5%	49.4%
UWLS‐RE	0	0.1	1.5	3	2.4%	51.9%
UWLS‐FE	0.25	0	0.5	5	2.3%	54.2%
UWLS‐FE	0	0	1.5	5	1.9%	56.1%
RE	0	0.1	1	5	1.9%	58.0%
RE	0.5	0.1	1	10	1.9%	59.9%
UWLS‐RE	0	0.1	1.5	5	1.8%	61.7%
RE	0.25	0.01	1	10	1.7%	63.4%
RE	0	0.01	1	5	1.7%	65.2%
RE	0.25	0.1	1	10	1.6%	66.8%
UWLS‐RE	0	0.01	1.5	3	1.6%	68.4%
UWLS‐FE	0.25	0	0.5	10	1.6%	70.0%
UWLS‐FE	0.5	0	0.5	3	1.5%	71.4%
UWLS‐FE	0	0	1.5	10	1.3%	72.7%
UWLS‐RE	0	0.01	1.5	5	1.3%	74.0%
FE	0.25	0	1	5	1.2%	75.2%
FE	0.25	0	1	10	1.2%	76.4%
UWLS‐FE	0.5	0	0.5	5	1.1%	77.5%
RE	1	0.1	1	10	1.0%	78.5%
FE	0.25	0	1	3	1.0%	79.4%
UWLS‐RE	0	0.01	1.5	10	0.9%	80.4%
UWLS‐RE	0.5	0.1	1.5	3	0.9%	81.2%
UWLS‐RE	1	0.1	1.5	3	0.8%	82.1%
UWLS‐RE	0.5	0.1	1.5	5	0.8%	82.8%
UWLS‐FE	0.25	0	1.5	3	0.7%	83.6%
RE	0.5	0.1	1	5	0.7%	84.3%
UWLS‐RE	0	0.1	1.5	10	0.7%	85.0%
UWLS‐RE	0.25	0.1	1.5	3	0.7%	85.6%
RE	0.25	0.01	1	5	0.7%	86.3%
RE	0.25	0.1	1	5	0.6%	86.9%
UWLS‐FE	0.25	0	1.5	5	0.6%	87.5%

*Note:* The table reports the top 40 DGPs by Mahalanobis weights. Each dataset in Stanley et al. was estimated using the UWLS‐RE estimator and the corresponding $\hat{\mu }$, $\hat{\gamma }$, and $\hat{\tau^{2}}$ values were recorded. Each set of $\hat{\mu }$, $\hat{\gamma }$, and $\hat{\tau^{2}}$ estimates was then measured for its distance from each of the 108 unique, triple values of *μ, γ*, and $\tau^{2}$ using the Mahalanobis distance function. In this way, each meta‐analysis dataset in Stanley et al. was matched with its closest DGP. Percentage weights represent the percent of meta‐analysis datasets in Stanley et al. that matched the respective DGP in the table.
